# Current status and future outlook of artificial intelligence and automation in intravenous infusion systems

**DOI:** 10.1038/s41746-026-03291-0

**Published:** 2026-10-14

**Authors:** Islam M. Ibrahim, Rahma A. Elkomy, Merna A. Mohamed, Fatemah A. Farok, Roaa E. Emam, Salma A. kamel, Reham M. Asem

**Affiliations:** Faculty of Engineering and Technology, Egyptian Chinese University, Cairo, Egypt

**Keywords:** Engineering, Health care, Mathematics and computing

## Abstract

The combination of the Internet of Things (IoT), artificial intelligence (AI), and wearable biosensors is transforming healthcare by allowing continuous, real-time monitoring and more intelligent clinical decision-making. This review explores AI-driven monitoring, smart intravenous (IV) infusion automation, and wearable biosensors, and proposes Intelligent IV Automation and Smart Patient Synchronization (INFUSYNC), a conceptual framework synchronizing monitoring, automated fluid delivery, and predictive analytics. The literature shows meaningful progress: wearables increasingly support non-contact monitoring with near clinical-grade accuracy for select parameters, while infusion pumps with dose-error-reduction and AI-assisted decision support are reported to improve delivery precision and safety. Predictive models further support early detection of physiological decline. Progress remains uneven, however: most AI-enabled infusion systems are prototype-stage, closed-loop control remains rare, and interoperability, data security, and privacy remain underdeveloped. Future systems will need compatible, security-by-design, clinically validated, feedback-controlled architectures. Overall, this review outlines a clear but early-stage trajectory toward integrated smart-health environments for IV therapy.

## Introduction

The arrival of the IoT, AI, and wearable sensors has fundamentally changed both the provision of healthcare as well as monitoring of patients^1,2^. Healthcare providers around the world are rapidly transforming how they look at individual health problems, track patient conditions, and provide care right from redesigning hospitals to rapidly developing diagnostics, drugs, and vaccines. This technological revolution provides an example of one of the most significant wholesale disruptive changes to modern medicine^3^. The disruptive nature of these kinds of technologies allows for ongoing, continuous, and real-time monitoring of patients, affording the healthcare system a new approach to longstanding problems in healthcare practice such as better resource utilization, patient safety, and decreased burden on healthcare workers. Contemporary healthcare delivery systems are grappling with increasingly complex problems that require innovative, technology-driven solutions ^4^. The sharp rise in healthcare infrastructure growth is representative of an increasing demand related to aging populations, number of people with chronic diseases, and number of patients with ongoing healthcare needs^5^. Likewise, the increasing complexity of surgical interventions necessitates advanced monitoring systems in order to have safe and effective care^6^. Healthcare professionals frequently treat patients experiencing multiple chronic conditions as opposed to just one condition, which reinforces the need for comprehensive monitoring systems which can record multiple physiological parameters, and provide early warning detection of abnormal values^7,8^. This is a shift from care being based on a reactive approach to proactive care supported through IoT-based real-time monitoring and timely intervention^9,10^. This is not only a technological step forward but wider engagement by the patient for prevention, early detection and personalized management of care. Not only are home and remote monitoring solutions extending the boundaries of patient care beyond the walls of the hospital^11,12^, but wearable biosensors have become the most fundamental element of contemporary patient management, whereby vital parameters can be universally monitored in a non-invasive and continuous manner which could not be done using traditional methods^13^. Advanced innovations which utilize Photoplethysmography (PPG) and Electrocardiography (ECG) methods within removable wearables have produced models which can reliably and reproducibly record Heart Rate (HR), Blood Pressure (BP), Respiratory Rate (RR) and Peripheral Oxygen Saturation (SpO₂) differentiation in wrist worn formats with clinically relevant accuracy^14,15^. Integration of several sensing technologies into one format has produced sophisticated monitoring systems, which ultimately can engage patients by providing complete health metrics in real-time^16^. The inclusion of AI and Machine Learning (ML) has broadened the functionality of wearable devices from basic data reporting to predictive analytics and the personalization of health data^17^. AI is capable of recognizing subtle patterns in data, which can indicate an early stage of health decline and allow for preventive action before symptomatic presentation^18^. In addition to monitoring, AI/ML are changing the ways decisions are made in the clinic, the efficiency of workers, and the management of patient’s lifestyles^19,20^. Nonetheless, major challenges will have to be overcome. These are accuracy of measurement across populations of patients, privacy and security of data and incorporation of wearable data into existing workflows in the clinic^21^. Motion artifacts, variation in skin tone, and differences in sensor calibration can all negatively affect data quality^22^. Health data must also ensure privacy and security provisions since this data can be so sensitive^23^. IV therapy is an important aspect of hospital care, with millions of patients receiving IV medications, fluids, and nutrients each day in health care environments throughout the world^24^. While conventional IV infusion systems work well, they are also defenseless to human error and rely on manual monitors, creating a significant burden for nurses and a risk to safety^25^. The increased complexity of drug protocols and infusions only worsens the possibility of dose and timing errors that can result in adverse drug reactions, failure of treatment, or life-threatening events^26,27^. Intelligent infusion pumps, which use Drug Error Reduction Software (DERS), drug libraries, and standardized alerts have improved dosing accuracy and patient safety^28,29^. These pumps have extensive medication databases that define concentration ranges, dosing limits, and compatibility standards, all in place to prevent the most common infusion errors^30^. In addition, with decision support tools embedded in the pump, the medication order can be processed in real-time, allowing the system to automatically detect and avert unsafe dosing^31^. Currently, smart infusion systems include automated monitoring and alert capabilities that reduce errors associated with improper doses, variations in infusion rates, and drug incompatibilities^32^. These infusion systems have now evolved to incorporate IV monitoring solutions that combine computer vision (CV), IoT sensors, and AI algorithms to analyse fluid levels, flow rates, line patency, and infusion site integrity^33–35^. IoT integration has enabled a shift in medical technology from stand-alone medical devices to connected medical devices that facilitate the efficient, transparent transmission of health information and validation of treatment across different care settings^36^. These systems will enable a shift from insurance of patient safety via stand-alone biomedical technology to real time interoperable subsystems and also to centralized computer monitoring stations^37^. The comprehensive patient monitoring across physiological parameters not yet achieved; however, connected and networked medical devices and sensors will help facilitate monitoring of patients in its entirety^8,38^. IoT-enabled healthcare solutions can bring together wearable sensors, alerting mechanisms, infusion pumps, and environmental sensors into a collaborative care delivery system^39^. The integration of data from multiple sources will provide a more comprehensive picture of the patient’s health and facilitate the identification of trends and anomalies not possible when looking at isolated data streams^40,41^. Typically, these systems take advantage of cloud computing, edge computing, and related mobile apps to allow real-time data retrieval and remote monitoring^12,42^. Cloud-based results promote clinician access to patient data from any location with an internet connection, while edge computing reduces delay and supports a more immediate response to emergencies^40,43^. AI and ML tools, effortlessly integrated into healthcare monitoring, allow systems that can analyse data, determine patterns, and create predictive models^20^. Specifically, ML algorithms process continuous physiological data, identify early signs of deterioration, and deliver clinical support with real-time analysis^44–46^. Likewise, CV applications have a high level of accuracy with automating monitoring tasks like IV bag identification, liquid level estimation, patient motion tracking and fall detection, which enhances clinician task performance while assuring consistency in monitoring^33–35^. While the technology has matured, it has not addressed all challenges. Data privacy, data security, and interoperability are still significant barriers to ubiquity^47–49^. The consequences of continuous monitoring increases new privacy issues as health data are often sensitive and personal^23^. There are also challenges related with interoperability between different medical devices, communication protocols, and data standards. The success of wearable biosensors relies on the adoption of shared standards, adherence to regulations and policies, consistent clinical trials to evaluate accuracy, reliability and usability among diverse populations and healthcare contexts^50–52^.

Despite the rapid growth of literature in each of these domains, existing reviews have largely treated them in isolation. Prior surveys on smart infusion systems^51^ have focused narrowly on pump automation and error reduction without integrating real-time patient physiological data. Similarly, reviews of wearable biosensors^52^ have concentrated on monitoring accuracy and device design without linking wearable output to therapeutic decision-making such as infusion rate adjustment. Reviews covering AI and IoT in healthcare more broadly^53^ provide useful taxonomies but stop short of addressing the specific workflow of IV therapy management. To our knowledge, no prior review has simultaneously synthesized wearable biosensor monitoring, smart IV infusion automation, and AI/ML-driven clinical decision support within a single unified framework. This review addresses that gap through the proposed INFUSYNC framework, which provides the first integrated perspective on how these three subsystems—continuous patient monitoring, intelligent fluid delivery, and data-driven analytics—can converge to form a safer, more responsive, and clinically viable healthcare monitoring system.

The paper is organized as follows: Introduction shows a brief to the current obstacles in healthcare delivery and the reason for integrated smart solutions. Review Methodology describes the structured approach employed to identify and review the relevant literature. Discussion presents an extensive view of the results, including general tendency, the relationship to the INFUSYNC framework, challenges and deployment readiness, and future research directions. Conclusion gives a summarizing key findings and proposed areas for future research.

## Review methodology

To represent the key technologies for future intelligent health systems, a three-tier methodological approach was adopted that examined the three component areas of IV infusion systems, wearable biosensors and AI/IoT delivery platforms within health care systems because of their interdependent nature and support for each other. Infusion systems provide accurate dosing of medication. Wearable devices enable continues monitoring of patients and AI/IoT technologies provide integration of patient information, automation of healthcare processes and decision-making. The combination of these technology domains was specifically targeted to determine how these will converge together to provide safe, timely, and patient-focused healthcare system.

## Eligibility criteria

The methodology in the selection process ensured reliability, relevance and quality of studies. The following content included utilizing predefined inclusion and exclusion criteria: next-generation IV infusion systems, wearable biosensors/bracelets, IoT-enabled healthcare apps, and AI-enabled patient monitoring systems (PMS).

## Inclusion criteria

Articles were included in this literature review only if they met all of the following criteria. Each criterion was deliberately selected to ensure that the reviewed literature directly supports the design and validation of the proposed INFUSYNC framework.

1. Time: Only articles published between 2020 and 2025 were included. This period was chosen because it reflects recent developments after Coronavirus Disease COVID-19, when technologies like IoT, AI, and wearable biosensors grew quickly in healthcare. This helps ensure that the studies represent current technologies, not outdated ones.
2. Quality: Only articles published in peer-reviewed journals or well-known conferences were included. This was done to make sure the studies are reliable and scientifically sound. Non-peer-reviewed sources cannot guarantee the same standard of independent validation and were therefore excluded to maintain the integrity of the review.
3. Domain: Articles must be related to at least one of the following areas:
  - Smart IV infusion devices and smart pumps.
  - Wearable biosensors and health monitoring bands.
  - IoT-based healthcare systems for real-time monitoring.
  AI-based healthcare applications focusing on accuracy, safety, ease of use, and prediction. These areas were chosen because they represent the main parts of the INFUSYNC framework. Studies outside these areas were not included, as they would not directly support the system design.
4. Measured Outcomes: Articles must report numeric or qualitative process monitoring outcomes related to at least one of the following:
  - Accuracy and reliability.
  - Patient and medication safety.
  - Medication errors and how to prevent them.
  - Ease of use and workflow improvement in clinical settings.
  - Health outcomes, cost impact, or system integration.
  This was required because studies with clear results can be compared more easily and provide stronger evidence. Studies without commensurable results were excluded.
5. Research Types: Both qualitative and quantitative studies were included:
  - Observational studies.
  - Clinical trials.
  - Systematic reviews.
  - Health economics studies.

Different types of studies were included to provide a complete view. For example, clinical trials show tested results, observational studies show real-world use, reviews combine findings from many studies, and economic studies help understand cost and practical use.

## Exclusion criteria

Articles were excluded if they fell within one of the following criteria. Each exclusion criterion was defined to prevent the studies that could compromise the scientific rigor, relevance, or reproducibility of the review findings.

1. Non-Healthcare Related Articles: Articles that were not related to healthcare applications were excluded, regardless of whether they involved relevant technologies such as IoT or AI systems.
2. Language Limitation: Non-English publications were excluded to maintain consistency in interpretation and avoid translation-related inaccuracies that could affect methodological and result validity.
3. Insufficient Empirical Validation: Theoretical studies without experimental evaluation, real-world deployment, or suitable methodological detail were excluded, as they do not provide verifiable evidence for system design or clinical applicability.
4. Low-Quality Publications: Editorials, commentaries, and conference articles abstracts lacking sufficient experimental or analytical data were excluded.

## Literature search and study selection

**Fig. 1:**
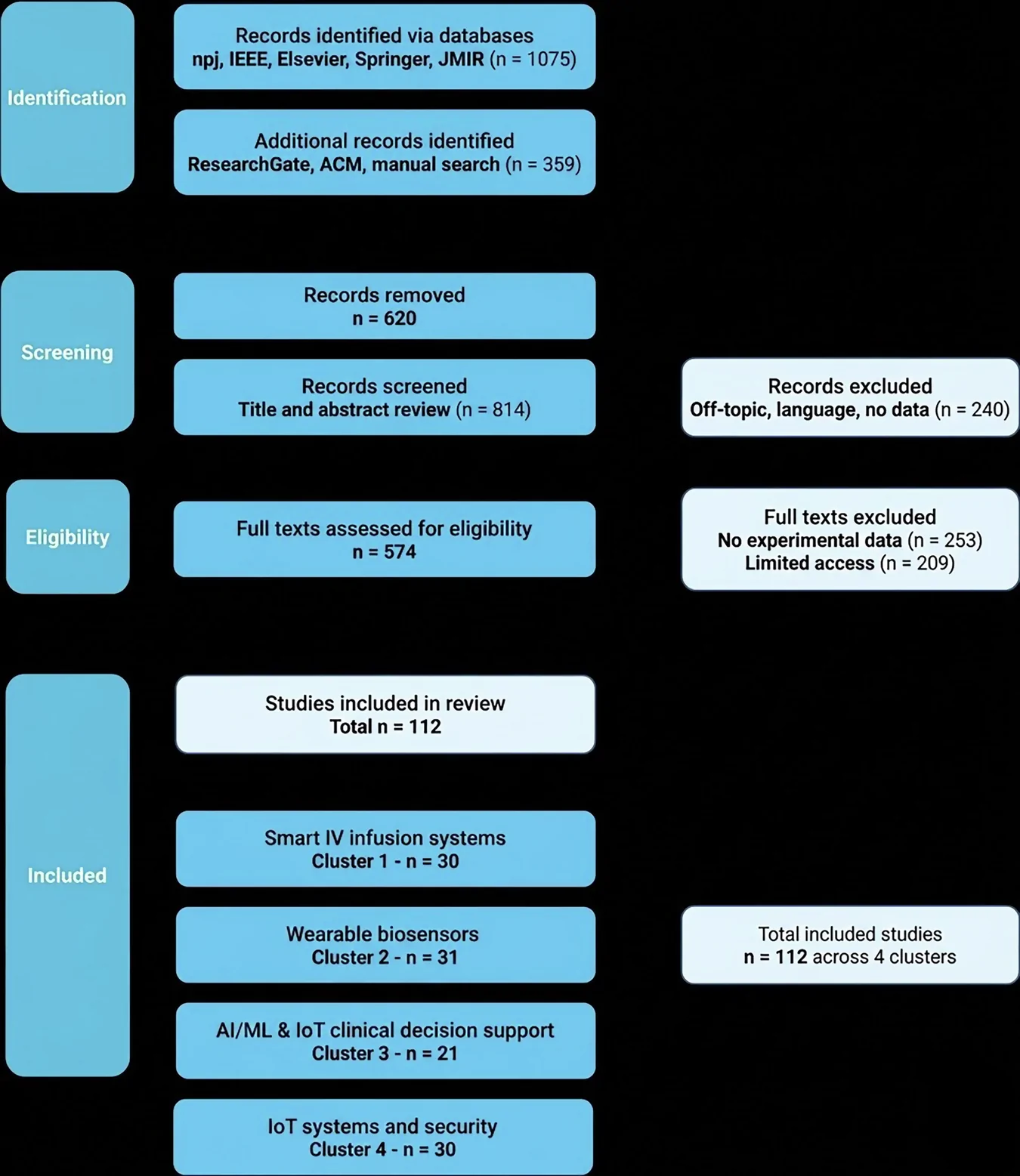
PRISMA flow diagram illustrating the systematic literature search, screening, and study selection process. Source: Created by the authors based on PRISMA guidelines. The diagram follows the PRISMA reporting standard and maps the stepwise selection of studies from an initial database search produce approximately 1075 records. Studies were progressively screened against predetermined inclusion and exclusion criteria covering publication year (2020–2025), peer-review quality, domain relevance, and measured outcomes. The final choice of 112 studies was distributed across four thematic clusters: smart IV infusion systems (30 studies), wearable biosensors (31 studies), AI/ML and IoT clinical decision support (21 studies), and IoT systems and security (30 studies). Each cluster matches to a core component of the proposed INFUSYNC framework. Records excluded at each stage are indicated with reasons, ensuring full transparency of the selection process.

A thorough investigation of published literature was performed utilizing journals of high prominence including npj Digital Medicine, Scientific Reports, Healthcare, JMIR, (Institute of Electrical and Electronics Engineers) IEEE Journal, Diagnostics, and Bioengineering, and databases or platforms including Springer, Elsevier, ACM Digital Library, and ResearchGate. The literature search focused primarily on recent studies and utilized the following five terms as search criteria: smart infusion pump, automated IV therapy, wearable biosensor, smart health monitoring, AI in healthcare, and IoT hospital systems. The use of these keywords was based on their intersection with each of the core areas encompassed by the INFUSYNC Framework. Figure 1 presents the Preferred Reporting Items for Systematic Reviews and Meta-Analyses (PRISMA) flow diagram summarizing the organized literature search and screening process, showing how the initial pool of approximately 1075 studies was gradually filtered to 112 eligible studies over four topical clusters that form the basis of the INFUSYNC framework.

## Data collection process and comparison

To support a clear and systematic review, the literature was collected and organized according to the four main parts of the proposed INFUSYNC framework: (1) IV infusion systems and automation, (2) wearable biosensors and smart bracelets, and (3) AI/IoT-based healthcare applications and clinical decision support. In each part, the studies were compared using clear criteria such as research aim, method used, application area, technologies involved, experimental design, results, and limitations. This approach helps to identify the strengths and gaps in each field and shows how these different tiers can work together. Overall, it supports the development of an integrated and patient-centered smart healthcare system.

## Wearable and IoT-based patient monitoring systems

Table 1 reveals that wearable biosensor research is predominantly review-based with restricted clinical deployment, as the majority of included studies are systematic reviews, scoping reviews, or narrative reviews rather than experimental or clinical validation studies. AI and ML integration is existing in fewer than a third of studies and is largely restricted to sensor-level processing or cardiovascular prediction rather than integrated clinical decision support. Real-time alerting capability is rare, and remote monitoring is almost entirely absent. Most critically, EHR integration, data security, and IV infusion system integration are entirely absent across all reviewed studies, representing the most significant gaps in current wearable biosensor technology. Table 2 presents the most representative studies to further illustrate these gaps.

**Table 1:** Comparative analysis of wearable biosensors and continuous patient monitoring

Refs.	AI/ML Integration	Real-Time Alert	Electronic health records (EHR) Integration	Remote monitoring	Clinical validation
^5^	No	No	No	No	Review paper
^6^	No	No	No	No	Review paper
^7^	Yes - ML	No	No	No	Review paper
^13^	No	No	No	No	Review paper
^14^	No	No	No	No	Prospective clinical study
^15^	Yes - in-sensor processing	Yes	No	No	Laboratory experimental
^16^	No	No	No	No	Review paper
^17^	Yes - AI multimodal	No	No	No	Review paper
^18^	Yes - AI bioelectronics	No	No	No	Review paper
^22^	Yes - ensemble ML	Yes	No	Yes	Experimental study
^50^	No	No	No	No	Systematic review
^51^	No	No	No	No	Scoping review
^52^	No	No	No	No	Scoping review
^96^	No	No	No	No	Review paper
^97^	No	No	No	No	Review paper
^98^	Yes - AI smart materials	No	No	No	Review paper
^99^	No	No	No	No	Review paper
^69^	Yes - CNN (Convolutional Neural Network)+ Deep Learning (DL)	Yes	No	No	Clinical validation
^100^	Yes - DL	No	No	No	Laboratory experimental
^101^	No	No	No	No	Review paper
^102^	No	No	No	No	Review paper
^103^	No	No	No	No	Mixed methods study
^104^	No	No	No	No	Survey study
^105^	No	Yes	No	Yes	Prototype
^106^	No	No	No	No	Review paper
^107^	No	No	No	No	Review paper
^108^	No	No	No	No	Review paper
^109^	Yes - AI cardiovascular	No	No	No	Review paper
^110^	No	Yes	No	No	Clinical perspective
^83^	No	No	No	No	Clinical validation
^70^	No	Yes	No	No	Clinical hospital study

Comparative analysis of wearable biosensor capabilities for continuous patient monitoring.

The following table provides the comparative study of wearable biosensor studies according to their AI incorporation, ability to provide real-time alerts, connection to electronic health record systems, remote monitoring facility, and clinical validation. Most of the studies have been conducted through reviews rather than any experiments or clinical implementations. AI incorporation is relatively uncommon in most of the studies; however, even where AI is incorporated, it is only through the simple analysis of the sensor data or predictive analysis. Real-time alerts and remote monitoring have been observed rarely in the studies, while the connection to EHRs has not occurred at all.

**Table 2:** Representative Wearable Biosensor

Refs.	Reason for selection
^14^	Best prospective clinical validation + real surgical patients, but no AI, no EHR, no IV integration
^15^	Best low-power in-sensor AI processing, but no EHR, no remote monitoring, no IV integration
^17^	Best AI multimodal wearable review, but no clinical validation, no EHR, no IV integration
^22^	Best ensemble ML with remote monitoring, but no EHR, no security, no IV integration
^69^	Best smartphone-based DL monitoring + clinically validate, but no EHR, no security, no IV integration
^110^	Best early deterioration detection perspective + clinical insight, but no AI, no EHR, no IV integration
^83^	Most innovative sensing modality + in-ear hemodynography + clinically validated, but no EHR, no security, no IV integration
^70^	Best acute hospital wearable validation + real clinical setting, but no EHR, no security, no IV integration

Representative wearable biosensor studies emphasizing key technological and clinical gaps.

The attached table highlights examples of high impact scientific studies that represent different levels of achievement within the field of wearable biosensors: examples include clinical validation; incorporation of artificial intelligence; and innovation in systems design and implementation. Regardless of the respective strengths of these independent studies, comprehensive systems integration is lacking across all of them. Studies which have been clinically validated lack intelligence derived from AI and interoperability; however, AI-orientated studies continue to be in either a ‘review’ or ‘test’ phase and are still not being implemented in the real world. The use of EHR’s; the implementation of security measures for sharing data; and the lack of integration with IV infusion systems are examples of elements common to each of these independent studies, regardless of their respective degrees of sophistication or complexity.

As demonstrated in Table 2, wearable biosensor research faces a parallel set of gaps to those identified in IV infusion systems. The most clinically validated wearable studies lack AI and hospital system integration, while the most AI-advanced studies remain at review or laboratory stage without clinical deployment. EHR integration, data security, and IV infusion system integration are completely absent across all reviewed studies regardless of technical sophistication. These findings mirror and reinforces the gap identified in the IV infusion cluster, confirming that no existing wearable system is designed to work in conjunction with IV therapy management. The INFUSYNC framework directly addresses this by suggesting the first unified architecture that connects continuous wearable patient monitoring with intelligent IV infusion control through an AI decision layer. Similarly, data security is entirely missing across reviewed wearable biosensor studies, despite wearable devices continuously collecting and transmitting highly sensitive physiological data including HR, SpO_2_, BP, and body temperature. The IoT healthcare literature provides validated solutions to this gap^12^: demonstrate the integration of AI and wearable technology within secure cloud frameworks for remote patient care^54^, propose AI-driven predictive analytics within secure wearable IoT architectures for real-time health monitoring, and^37^ provide a comprehensive review of IoT-assisted wearable sensor systems with explicit discussion of security requirements. At the system integration level^41^, propose a secure cloud-based precision health system for IoT big data, while^3,10^ provide comprehensive reviews of key IoT healthcare technologies including security frameworks applicable to wearable data pipelines. The fact that none of these security approaches have been translated into wearable biosensor system design confirms that security remains a critical unaddressed gap in the wearable monitoring literature, and that INFUSYNC must incorporate privacy-preserving data transmission as a foundational design requirement for its wristband monitoring module.

## Smart intravenous infusion systems

Analysis of Table 3 exposes that while real-time alerting is the most commonly implemented feature, the majority of reviewed IV infusion systems remain at prototype stage with limited clinical deployment. AI integration where present is largely limited to basic detection rather than intelligent decision support, and most systems operate individually rom hospital information systems. Most critically, data security and wearable patient monitoring integration are rare across all reviewed studies, which represent the most significant gaps in current IV infusion technology. Table 4 presents the most representative studies to further illustrate these gaps.

**Table 3:** Comparative analysis of smart IV infusion systems

Refs.	AI/ML integration	Real-time alert	EHR interoperability	Remote monitoring	Clinical validation
^24^	No	No	No	No	Comprehensive review
^25^	No	Yes	No	No	Design study
^26^	No	No	Partial	No	Scoping review
^27^	Yes -theoretical AI	No	Partial	No	Expert panel - theoretical
^28^	No	Yes	Yes - HL7/FHIR (Health Level 7 Fast Healthcare Interoperability Resources)	Yes	Health economic analysis
^29^	No	Yes	Yes -interoperability review	No	Systematic review
^30^	No	Yes	No	No	Prototype - Scientific Reports
^31^	Yes - ML anomaly detection	Yes	No	No	Retrospective hospital dataset
^32^	Yes -context-aware AI	Yes	Yes -bidirectional	No	Prototype/conference
^33^	Yes DL-CV	Yes -automated	No	No	Experimental validation
^34^	Yes - semantic segmentation	Yes - automated	No	No	Experimental -Sensors
^35^	Yes -DL-CV	Yes -automated	No	No	Experimental
^53^	No	No	No	No	Systematic review
^56^	No	Yes	Yes - EHR integration	Yes	Clinical ICU (Intensive Care Unit) study
^59^	No	Yes	Yes - smart pump EHR	No	Clinical ICU study
^89^	No	Yes - real-time	No	Yes	Prototype
^55^	Yes - closed-loop DL	Yes - automated	No	No	Experimental
^92^	No	Yes	Yes - pediatric	No	Clinical - pediatric hospital
^90^	No	Yes - real-time	No	Yes	Prototype
^57^	No	Yes - real-time	No	Yes	Retrospective hospital study
^84^	No	Yes -buzzer/alert	No	No	Prototype - Arduino
^85^	No	Yes - real-time	No	No	Prototype
^86^	No	Yes	No	No	Prototype
^111^	Yes - hybrid ML	Yes - IoT cloud	No	Yes	Simulation/experimental
^58^	No	Yes - centralized	No	Yes	Cross-sectional - ward study
^87^	No	No	No	No	Experimental
^88^	No	Yes - capacitive	No	No	Experimental
^68^	No	Yes	No	Yes	Prototype
^91^	No	Yes - mobile alert	No	Yes	Prototype
^112^	No	Yes - flow imaging	No	No	Experimental

Comparative analysis of smart IV infusion systems and monitoring capabilities.

This table presents a comparison between representative smart IV infusion systems according to the extent of AI integration, real-time alerting capabilities, EHR connectivity, remote monitoring abilities, and level of clinical validation. Real-time alerts represent the most common feature among all systems, whereas the majority of the existing systems are still experimental prototypes with no real-world application. Most applications of AI techniques include anomaly detection or computer vision approaches for monitoring purposes but not for advanced decision-making support. The number of papers describing integration into the hospital environment is low, and the number of studies that support interconnectivity of the smart IV system with EHR through HL7/FHIR is even lower. The most crucial limitations include the lack of reliable communication between the device and the physician and no integration with continuous monitoring devices, e.g., wearable devices.

**Table 4:** Representative smart IV infusion systems

Refs.	Reason for selection
^27^	Only paper proposing theoretical AI for smart pumps, proves AI still not implemented.
^31^	Only ML-based alert detection with real hospital data, but no EHR, no remote monitoring, no wearable.
^33^	Best CV-DL for IV bag, but no EHR, no remote monitoring, no clinical validation.
^56^	Best EHR interoperability + remote monitoring + clinical ICU, but no AI, no security.
^59^	Real ICU clinical evaluation with EHR, but no AI, no remote monitoring, no security.
^55^	Only closed-loop AI system, but no EHR, no remote monitoring, no clinical validation.
^57^	Best IoT remote monitoring with real hospital data, but no AI, no EHR, no security.
^58^	Only real ward deployment with remote monitoring, but no AI, no EHR, no security.

Representative smart IV infusion systems show key technological and clinical gaps.

This table presents selected unbiased sample highlighting key strengths and limitations of current smart IV infusion systems, including artificial intelligence capabilities, clinical validation, system interoperability, and monitoring functionality. While some systems demonstrate clinical deployment and EHR integration, they lack intelligent automation and security mechanisms. AI-based systems have advanced analytical capabilities; however, they are not commercially available and only exist as test or prototype systems, which have not been validated in real life. All of the studies reviewed demonstrated that many of the critical elements to implement these systems in the real world are missing from the design of current systems, including the secure transfer of data, the integration of wearable patient monitoring into these systems, and the complete automation of closed loop infusion control.

As demonstrated in the representative analysis Table 4, a fundamental trade-off exists across current IV infusion systems: clinically validated systems lack AI, while AI-advanced systems lack clinical validation. Furthermore, data security and wearable patient monitoring integration are entirely absent across all reviewed studies regardless of their technical sophistication. This gap confirms that no existing IV infusion system simultaneously achieves clinical validation, AI-driven intelligence, secure data transmission, EHR interoperability, and integration with continuous patient monitoring. AI and ML are progressively used to enhance IV infusion safety by detecting medication errors earlier than traditional rule-based systems^31,55^. However, many of these approaches are still limited, as most AI-based systems lack strong real-world clinical validation and rely on scanty infusion datasets^27^. A major gap also exists in closed-loop control, where most systems can detect problems but still require manual clinician intervention instead of automatically adjusting infusion rates^56–58^. In addition, interoperability with hospital systems is weak, with very few solutions integrated with EHR or compliant with HL7 FHIR^56,59^. Overall, this appear that IV infusion automation still requires further development in AI-based detection, closed-loop control, and system integration to match the attention given to wearable devices.

Data security, an important aspect lacking adequate coverage in the reviewed IV infusion literature. Though some studies assume safety data processing is critical in the therapeutic setting, documentation of resilient and methodically implemented security protections within IV infusion systems themselves are inconsistent or entirely absent. Such problems are more significant considering the sensitivity of medication administration data relayed between infusion pumps, monitoring systems and hospital network. In contrast, the more extensive IoT healthcare domain has explained specific implementation in developing and validating robust data protection solutions. These include blockchain-enabled federated learning (FL) frameworks for secure data sharing^50^. Authorized blockchain architectures drafted for energy-efficient and secure healthcare IoT systems^60^, blockchain-secured EHR for smart healthcare environments^61^, and partitioned blockchain encryption approaches that enhance privacy in IoT-based medical applications^62–64^. Constantly recognize security and privacy as persistent and critical challenges across IoT healthcare deployments, while^65,66^ provide a comprehensive taxonomy of blockchain-based solutions tailored for AI-driven healthcare systems. This limited adoption of deep-rooted security approaches emphasizes a clear gap between existing technological competencies and their practical application in IV infusion systems. To bridge this gap, there is a need to move beyond treating security as a secondary consideration and instead adopt a security-by-design approach, where data protection is integrated as a core element of system architecture from the outset.

## Visualization and data representation

This section includes visual representations and diagrams of the three tiers of the INFUSYNC system IV infusion systems, wearable biosensors, and healthcare applications with AI/IoT-based to help provide clarity. The structures, functionalities, design, and results of the relevant technologies described in the literature were synthesized by visual representation for each relevant technology category.

**Fig. 2:**
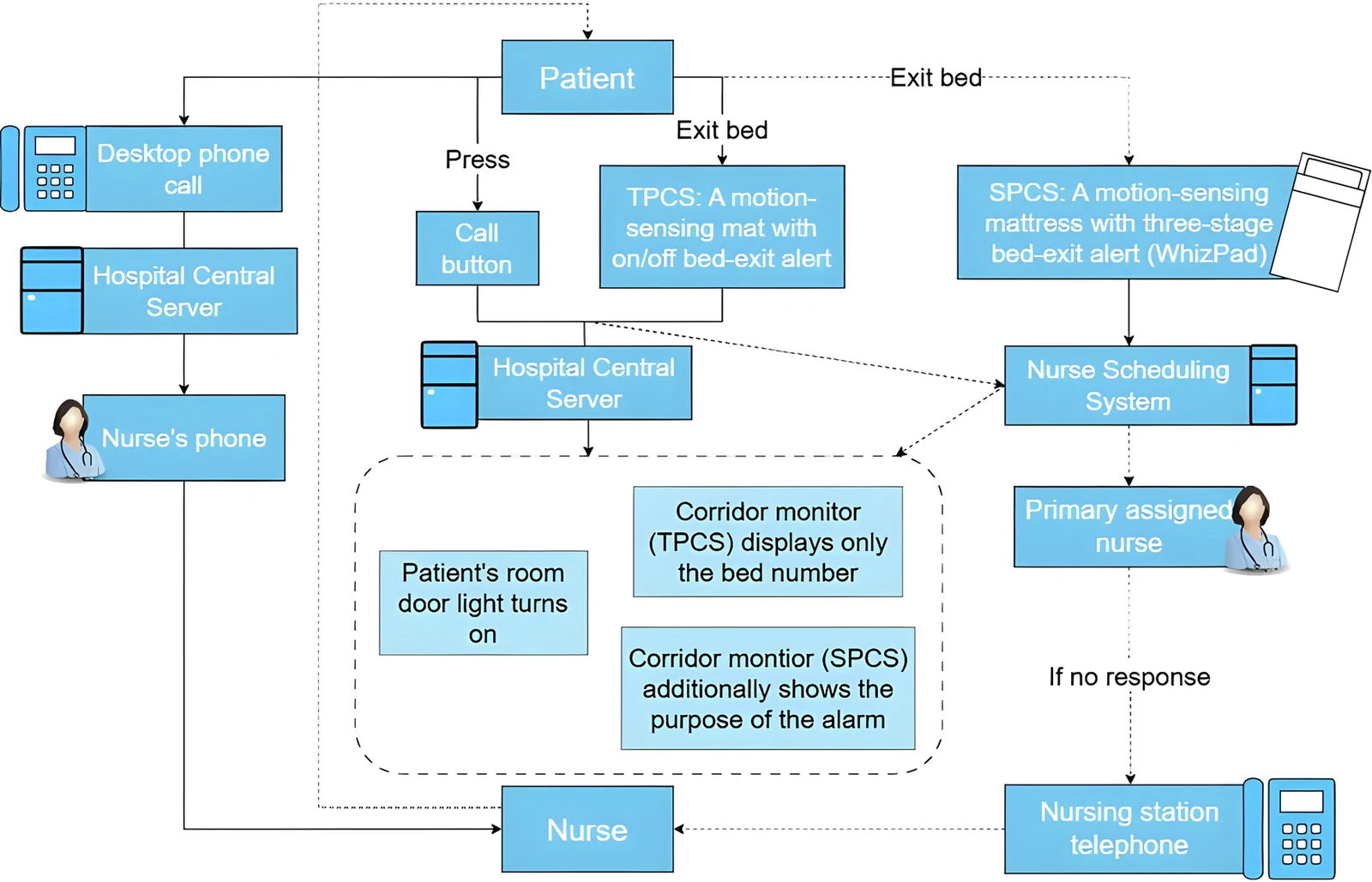
Comparative illustration of a traditional patient care system and a smart patient care system in a hospital ward, demonstrating enhanced alert classification and nursing response capability in the smart system^2^. The two systems being referred to here are a TPCS and a SPCS. When a patient calls for assistance, they are using one of the buttons on the system to trigger an alert, including the patient’s bed number, which is displayed on the monitors in the hallway and also at the Nursing Station. One key difference between the TPCS and SPCS is that only the bed number is displayed with the TPCS and there is no other information provided about that patient or their condition. Conversely, the SPCS will show both the bed number and the type of alert associated with the request. Because the SPCS has added the alert type classification element to the system, the nursing staff can now prioritize the urgency of their response, thereby reducing the number of unnecessary emergency response calls for routine assistance requests and more quickly respond to actual emergency situations. There was a measurable improvement in both the overall efficiency (work flow) and in response accuracy as the result of utilizing the SPCS versus TPCS in a live clinical ward evaluation. The intelligent alerting system component that is found within the SPCS is part of the INFUSYNC framework and represents a key building block in the continued development of an IoT-based nursing support network.

Figure 2 compares Traditional Patient Care System (TPCS) with Smart Patient Care System (SPCS) in hospital wards for handling nurse calls and alerts ^2^.

The SPCS, introduced in a hospital ward in 2017, was compared with the TPCS as shown in Fig. 2. In both systems, when a patient clicks the call button, an alert is triggered and the bed number appears on monitors in the corridor and nursing station. The major difference is that the SPCS also present the type of alert (such as normal, emergency, or bed-exit), which helps nurses understand the situation and take action more quickly and appropriately^2^.

**Fig. 3:**
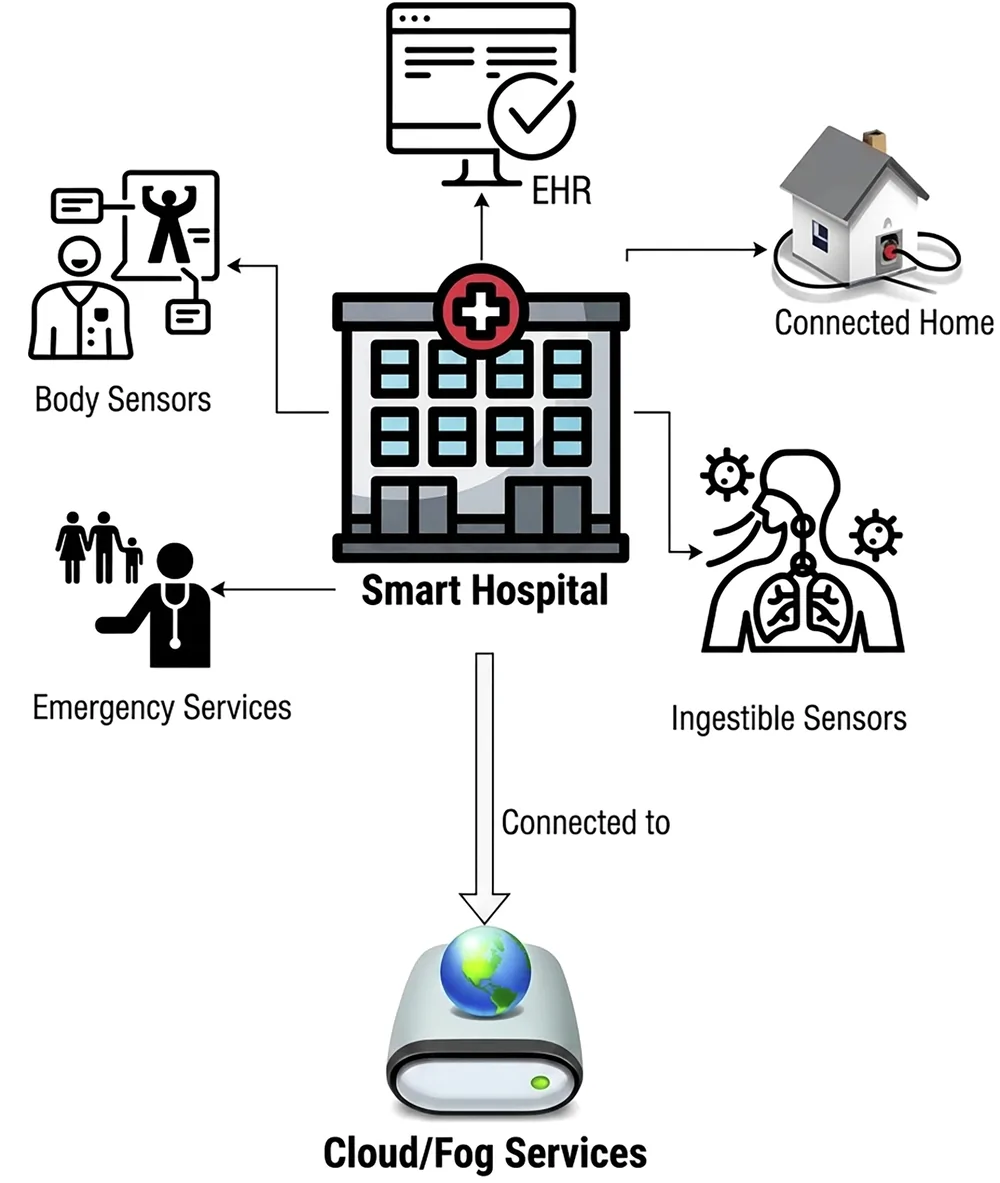
Conceptual overview of an IoT-assisted smart hospital ecosystem illustrating real-time patient data collection, transmission, and integration with hospital information systems and electronic health records^37^. The figure portrays a comprehensive smart hospital environment in which multiple IoT sensing modalities operate in parallel. Body-worn sensors and ingestible sensors continuously capture patient physiological parameters, including HR, oxygen saturation, and body temperature, while environmental sensors monitor ward conditions. All collected data streams, whether they initiate from a hospital’s central information system or a remote hosted platform, get sent into a recording system called EHR. In this way, the information gets continuously monitored as the patient is being treated in the hospital. The design of the system provides healthcare providers with the ability to obtain real-time clinical data about their patients, whether they are located at the bedside or remotely. Therefore, the provider is able to make clinical decisions in a timely manner and effectively reduce information latency.

Figure 3a smart hospital uses IoT sensors to continuously collect patient data in real time and send it to hospital systems or EHRs for monitoring and diagnosis^37^.

A smart hospital ecosystem combines a range of IoT technologies to ensure efficient and real-time healthcare services shown in Fig. 3. Body sensors and ingestible sensors will capture patient health data continuously in real-time, with the data being delivered to the hospital system, or remotely located system, and entered in EHR for continuous monitoring and diagnosis^37^.

**Fig. 4:**
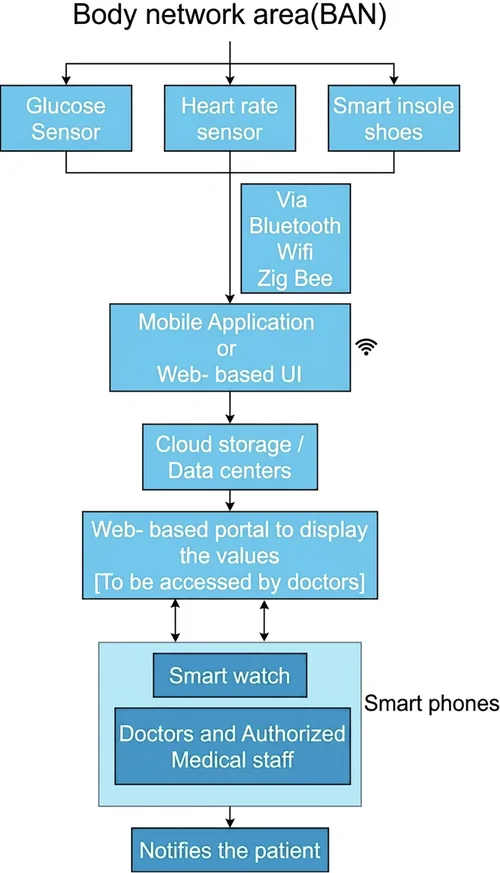
Architecture of a body area network-based IoT health monitoring system showing wearable sensor data transmission through a smartphone application to a cloud platform for remote physician access and automated alerting^37^. As depicted in the figure, there is an architectural diagram where the communication process occurs using layered structure where wearable sensors are applied on the patient’s body to collect information about various physiological parameters such as sugar content in the blood, HR, and physical activity. The sensor data collected is sent through either Bluetooth or Wi-Fi connection to the mobile app, which serves as a data aggregator as well as interface for the user. From the phone, data is sent to cloud storage processing center for further processing and analysis and also to be accessible by the remote doctors. There is an automated alert facility provided by the monitoring tool.

Figure 4 illustrates a body area network (BAN)-based IoT monitoring system in which patient sensors transmit glucose, HR, and activity data via Bluetooth/Wi-Fi to a smartphone app and cloud platform, enabling real-time physician access and abnormal reading alerts^37^.

**Fig. 5:**
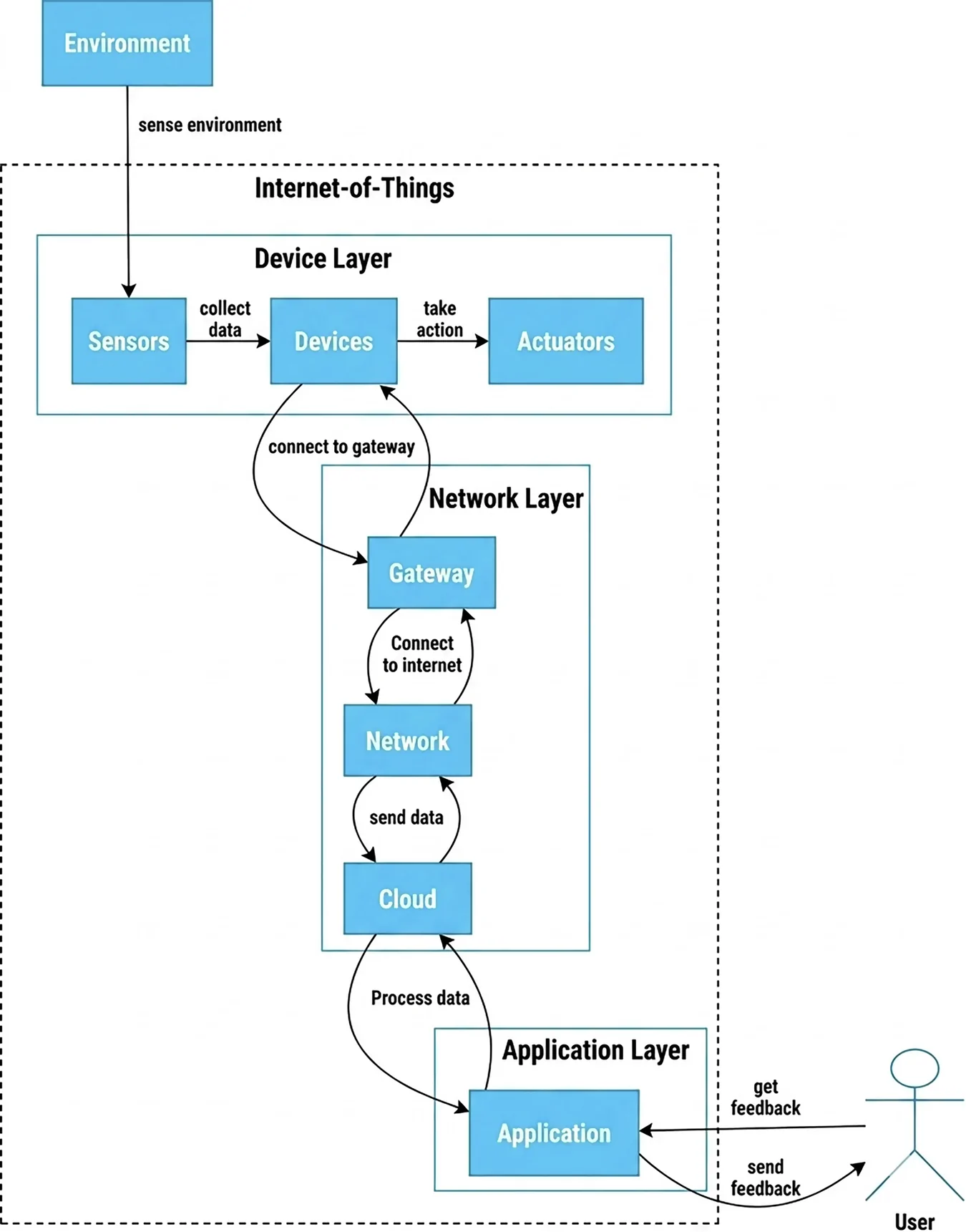
Flowchart of the generic three-layer Internet of Things architecture applicable to smart healthcare systems, comprising device, network, and application layers^67^. Layer Device at the bottom of the architecture comprises physical sensors and wearable devices used to capture physiological and environmental data about the patient or healthcare setting. The generated data is then forwarded to the network layer, from which it is delivered safely to the cloud servers through communication channels like Wi-Fi, Bluetooth, and cellular networks. Above the architectural hierarchy is the Application Layer in which algorithms and artificial intelligence techniques are employed to interpret the data and derive valuable clinical information from it. For smart healthcare applications, the three-layer structure provides constant monitoring, predictive analysis, and feedback mechanisms among others. The INFUSYNC system has been designed around this architecture where the three subsystems within it – wearable monitoring, infusion control, and AI decision support – correspond to layers.

The architecture of Fig. 5 illustrates layered structure enables efficient real-time monitoring, intelligent decision-making, and automated control in smart healthcare systems^67^.

Figure 5 shows a simple three-layer IoT architecture made up of the device, network, and application layers. The device layer collects real-time data using sensors and wearable devices, while the network layer is responsible for sending and securing this data through technologies like Wi-Fi, Bluetooth, and cloud systems. The application layer then processes the data using AI to provide useful insights, support decision-making, and enable automation in fields like smart healthcare^67^.

**Fig. 6:**
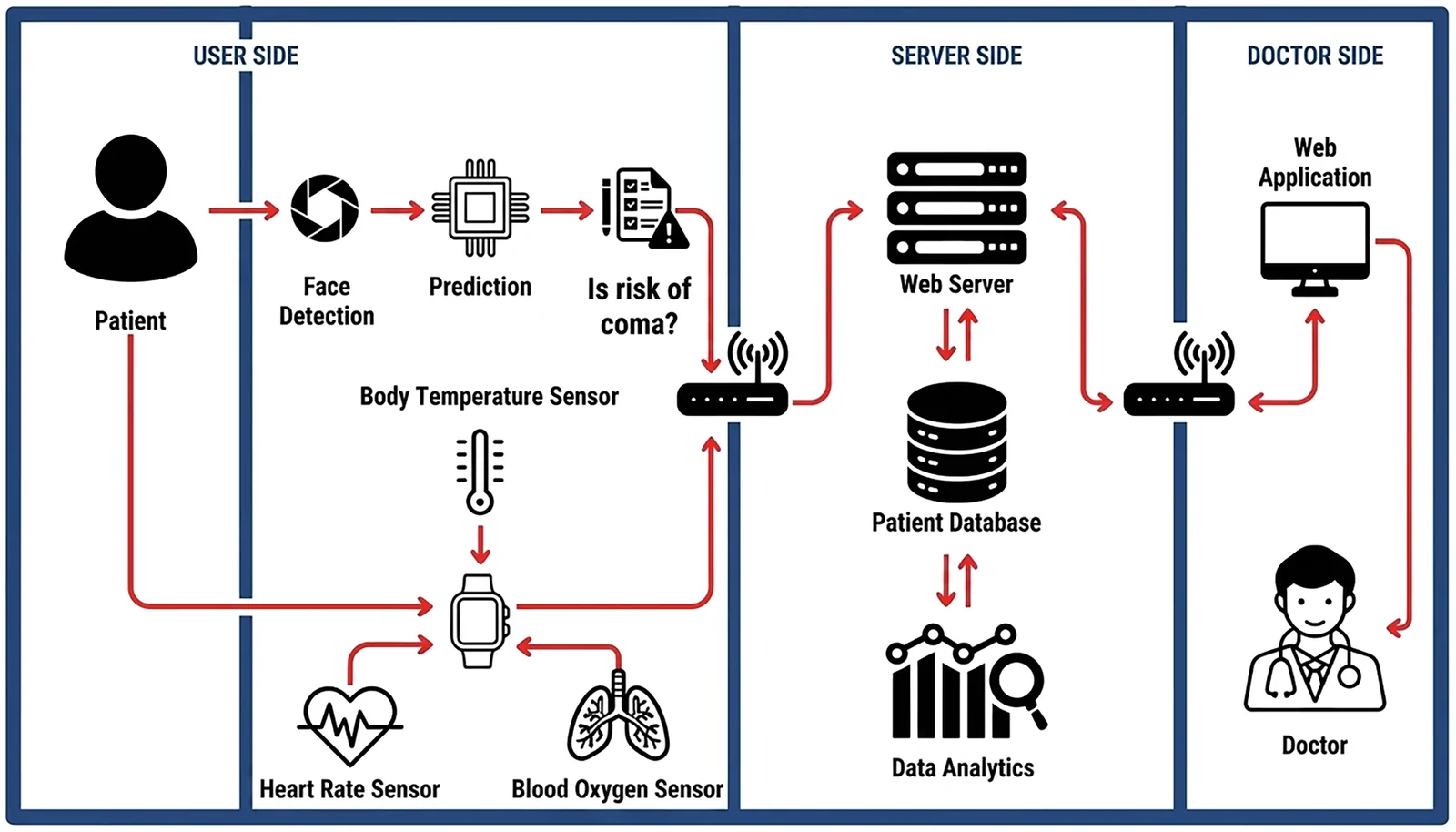
System architecture of an IoT-based wearable health monitoring platform integrating physiological sensing, facial emotion recognition, cloud storage, and AI-driven real-time health risk prediction^54^. Three physiological factors—including HR, peripheral oxygen saturation (SpO2), and body temperature — are collected via wearable sensors every minute. In addition, the facial expression captured by a camera equipped with artificial intelligence is analysed to determine the patient’s mood status. The data collected from sensors as well as pictures is transmitted using Wi-Fi technology into the cloud database. The cloud database will then be used for storing, classifying, and making the database accessible through the user interface of the web application. The AI analytics component analyses the incoming data stream to measure the health risk of the patients and raise alarms upon identification of irregularities and deteriorating trends. Medical practitioners and hospital management can monitor the health status of the patient in real-time via the web application interface.

Figure 6 presents an IoT-based wearable system that constantly collects patient vitals and facial data, send them to a cloud database, and analyzes them using AI models to predict health risk in real time^54^.

As shown in Fig. 6 the system uses wearable sensors to measure HR, SpO₂, and body temperature every minute, while an AI camera is used to take facial emotions. All the collected data is transmitted through Wi-Fi to a database, where it is stored and managed, and then displayed on a web application. The system analyzes patient data to check risk, sends alerts when needed, and lets medical staff and administrators monitor patients in real time^54^.

**Fig. 7:**
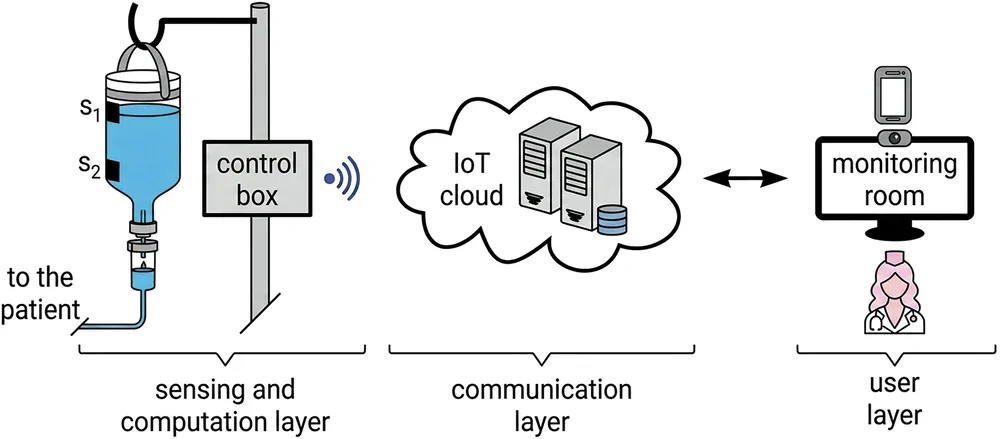
Layered architecture of an IoT-based smart IV infusion monitoring system allowing real-time remote fluid level surveillance and automated clinical alerts^68^. Two sensors (S1 and S2) positioned on the infusion bottle continuously detect fluid levels and transmit the collected data to a bedside control unit for initial processing. The control unit then relays the data wirelessly to an IoT cloud platform, where the information is stored and made accessible remotely. Healthcare staff can monitor infusion status in real time from any location with network connection and receive automated alerts when fluid levels fall below a determined threshold, assure timely intervention and uninterrupted patient care. This layered design reflects the standard three-layer IoT architecture applicable to smart healthcare deployments.

Figure 7 shows an IoT-based infusion monitoring system where data transmitted to a control unit and cloud platform enables real-time remote monitoring and alerts for medical staff, improving responsiveness and patient care^68^.

Figure 7 shows a system where sensors (S1 and S2) inside an infusion bottle that detect the fluid levels. These sensors send the data they collect to a control box, which then processes the information. After that, the control box uses wireless transmission to send the data to the IoT cloud, in which it can be monitored in real-time. This way, healthcare staff can access the system from wherever they are. They’ll get alerts when the fluid levels are running low, so they can make sure patients are always getting the care they need without any interruptions^68^.

**Fig. 8:**
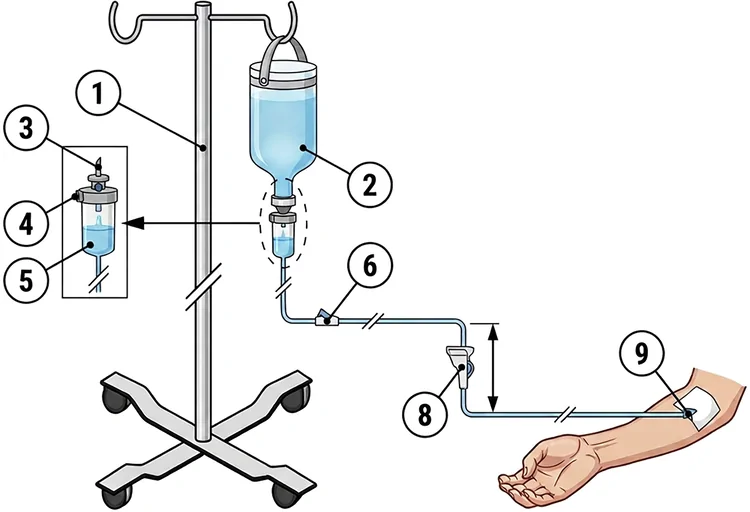
Annotated schematic of the standard components of a conventional IV infusion therapy set^68^. The figure identifies nine core components that collectively regulate and deliver IV fluids to the patient. (1) the stand that supports, (2) the IV bag containing the infusion fluid, (3) the spike, connects the IV bag to (7) the IV tubing, which transports the fluid toward the patient, (4) an air-release valve that prevents vacuum formation within the line, (5) the drip chamber, enables visual monitoring of fluid flow and drip rate, (6) a secondary infusion site that allows supplementary medication to be introduced into the line. Fluid flow through the tubing is regulated by (8) the roller clamp, (9) the cannula, is inserted into the patient’s vein to deliver fluid directly into the bloodstream. Together, these components form the foundation of traditional IV therapy delivery, providing the anatomical context for understanding the automation and sensing innovations discussed in this review.

Figure 8 illustrates the standard components of an IV infusion set, these components ensure controlled fluid allow monitoring and regulation of the infusion process^68^.

As shown in Fig. 8 an IV infusion set typically includes several standard components that function together in order to provide fluids to your patient through IV therapy. The components of an IV infusion set are: **(1) the stand supporting** the **(2) IV bag**, and connected via the **(3) spike** to the **(7) IV tubing**; **(4) a valve** to release air to avoid the formation of a vacuum; **(5) drip chamber** to monitor fluid flow and observe drip rate; **(6) a secondary infusion site** to accept additional medication if needed; and **(7) IV tubing** to transport the fluid, which is controlled by **(8) roller clamp**; and **(9) the cannula** inserted into the patient’s vein to ensure fluid is directly inserted into the blood stream^68^.

**Fig. 9:**
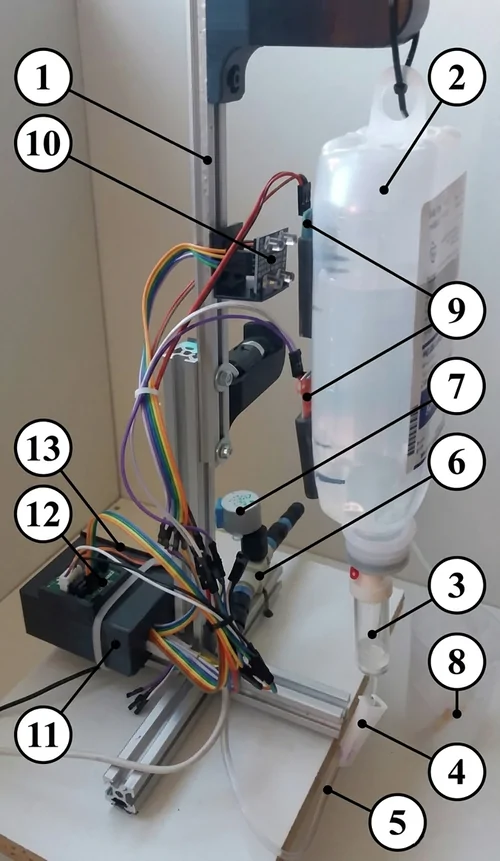
Hardware schematic of an intelligent infusion bottle tracking system incorporating capacitive level sensing, color identification, wireless communication, and automated flow control^68^. The system comprises thirteen labeled components working in coordination to track and control IV fluid delivery. The stand (1) sustains the saline bottle (2), drip chamber (3), and roller clamp (4), which are connected via IV tubing (5). A valve (6, type VHK2-08F-08F) and stepper motor (7, model 28BYJ-48) enable automated flow control, while the cannula (8) delivers fluid to the patient. A capacitive level sensor (9) compatible with Arduino detects the remaining fluid volume in the bottle, and a color sensor (10, model TCS230) identifies fluid type or concentration by optical detection. System handling and wireless communication are managed by a microcontroller (11, Arduino Uno) paired with a Wi-Fi module (NodeMCU ESP8266), enabling real-time remote monitoring and alerts. A motor driver (12, model 2PH64011A) controls the stepper motor, and an LCD display (13, model LCD12864) provides local status readout. This integrated design minimizes manual monitoring requirements, prevents infusion interruptions, and enhances patient safety through automated detection and signaling.

Figure 9, Table 5 illustrates an intelligent infusion monitoring device that uses multiple sensors to detect liquid levels and automatically alert users in real time ^68^.

**Table 5:** Description of the components in the liquid level detection and signaling system for infusion bottles shown in Fig. 9

No.	Part
1	Pole/stand
2	Saline bottle
3	Drip chamber
4	Roller clamp
5	IV tubing
6	Valve VHK2-08f-08f
7	Stepper motor 28BYJ-48
8	Cannula
9	Capacitive level sensor for Arduino
10	Color Sensor TCS230
11	Microcontroller Arduino Uno and Wi-Fi module NodeMcu ESP8266
12	Motor driver 2PH64011A
13	Liquid Crystal Display (LCD) 12864

Hardware components of the intelligent IV infusion monitoring and control system.

This table lists the main hardware elements used in the proposed infusion monitoring device, including sensing units, control modules, and mechanical components. The system integrates capacitive level sensing for fluid monitoring, color-based detection for flow verification, wireless communication modules for remote data transmission, and a stepper motor-driven mechanism for automated flow control. These components together allow real-time monitoring and partial automation of IV infusion processes, reducing manual intervention and supporting clinical efficiency.

As shown in Fig. 9, the system presents an intelligent infusion monitoring device that automatically detects and alerts users about liquid levels in infusion bottles. It integrates capacitive sensors for level detection, color sensors for fluid identification, and a microcontroller with wireless communication for real-time monitoring and alerts, along with a step motor and valve for flow control. This combined design helps reduce manual monitoring, prohibit infusion interruptions, and enhance patient safety. Table 5 describes each numbered device in the schematic. This combined design can be used for consistent idle prevention and minimized monitoring while increasing patient safety^68^.

**Fig. 10:**
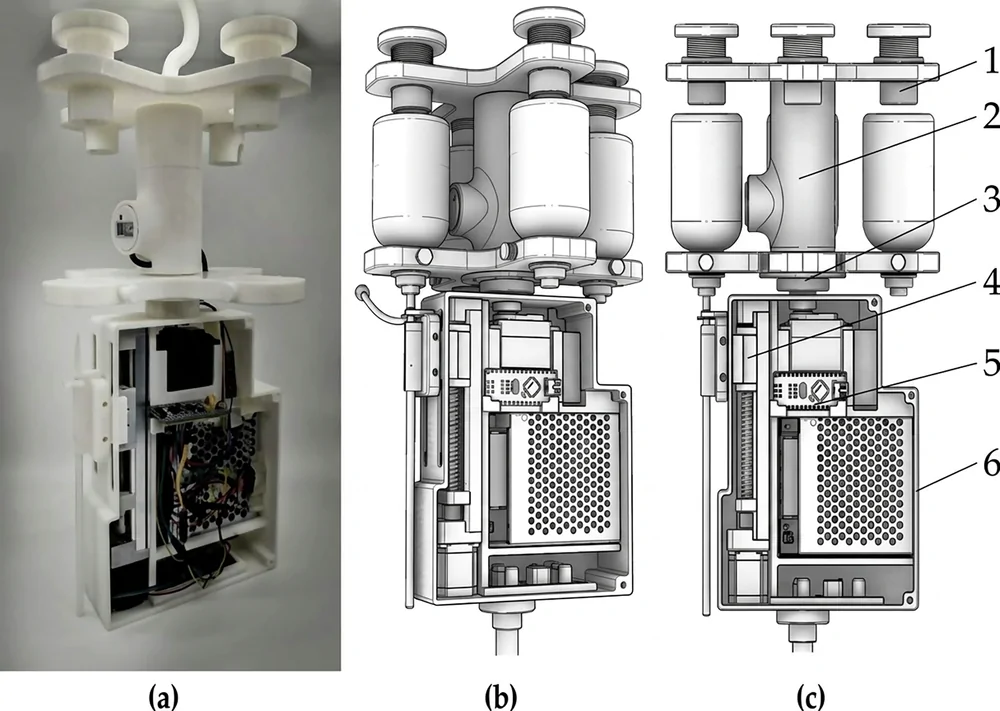
Smart infusion automatic medication replacement device showing the physical prototype and annotated structural model with six labeled functional subsystems^30^. **a** Shows a photograph of the actual prototype device as constructed and tested. **b**, **c** Present front and side structural model diagrams with six core functional subsystems identified and labeled. Subsystem 1 is the fixed calibration unit, which positions the infusion containers with precision. Subsystem 2 is the liquid level monitoring module, which continuously tracks remaining fluid volume. Subsystem 3 is the rotational replacement system, facilitating automated transitions from one medication bottle to another without human interaction. Subsystem 4 is the plug-and-push mechanism responsible for piercing and engaging successive infusion containers. Subsystem 5 is the control module, which manage all subsystem operations based on sensor inputs. Subsystem 6 represents the external casing that contains the entire device and holds the various inner parts in place and acts as a shield against any damage to the subsystems. Once all the subsystems combine to form the system, the automatic delivery of the drug through IV would not be hindered by the intervention of a nurse when the bottle empties itself.

Figure 10 illustrates a smart infusion device that integrates real-time fluid monitoring with automated medication replacement to improve IV therapy efficiency and accuracy, reduces nurse workload, minimizes human error and enhance patient safety^30^.

Figure 10 shows the smart infusion automatic medication replacement device, including (a) the actual photograph of the device and (b) the model diagram of the system. The design contains six main components: **(1) fixed calibration, (2) liquid level monitoring, (3) rotary replacement, (4) plug-and-push, (5) control module**, and **(6) device casing**. The system is designed to automate IV medication replacement, reduce manual workload, and enhance dosing accuracy and patient safety^30^.

**Fig. 11:**
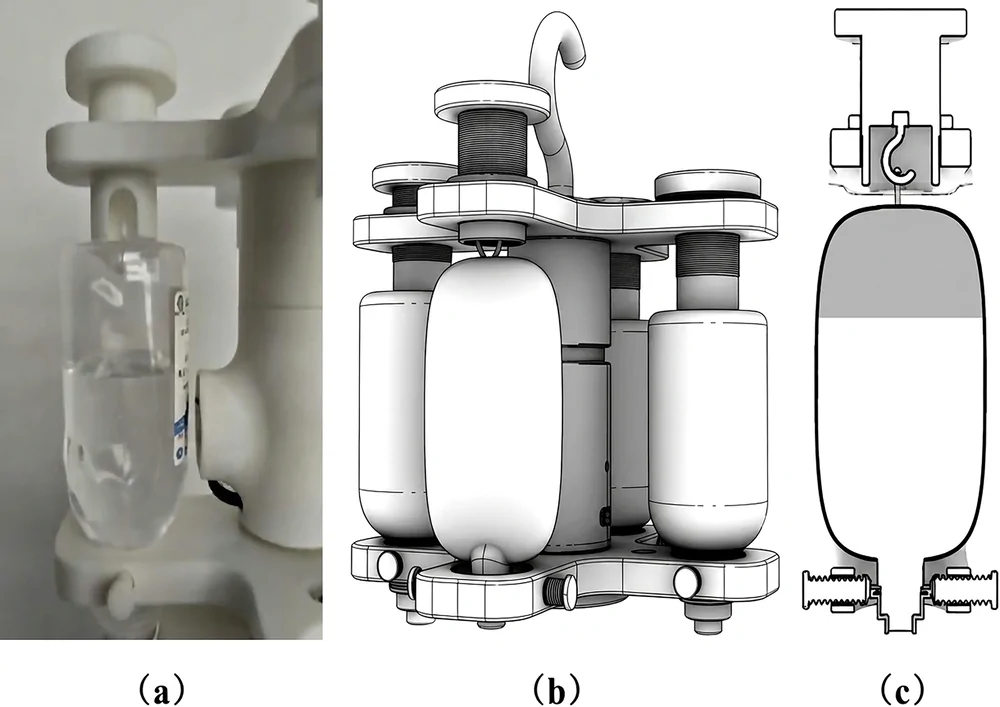
Medicine bottle and infusion bag stabilization system showing the physical device, installation configuration, and cross-sectional fixation mechanism^30^. **a** Shows a photograph of the stabilization device as physically implemented. **b** Presents the installation model demonstrating how the system is mounted within the infusion apparatus. **c** Provides a cross-sectional schematic detail of the fixed mechanism at both the base and body of the infusion container. The system uses hooks and fixed anchor points to hold medicine bottles and infusion bags securely in position throughout the infusion cycle, preventing tipping, accidental disconnection, or lateral displacement during automated bottle-switching operations. By sustaining stable container positioning, the device ensures consistent gravitational fluid flow, reduces the risk of flow interruption caused by mechanical disturbance, and contributes to overall patient safety during automated IV medication replacement procedures. This stabilization mechanism operates in conjunction with the rotary replacement and plug-and-push subsystems described in Fig. 10.

Figure 11 presents a stabilization system that secures infusion bottles to prevent displacement during suctioning ensures consistent fluid flow, reduces interruptions^30^.

Figure 11 shows a stability system for medicine bottles and infusion bags that uses hooks and fixation points at the base and body to keep them securely in place during infusion. This design prevents accidental movement, tipping, or disconnection of the containers and helps maintain stable and continuous infusion flow. As a result, it enhances infusion consistency and enhances overall patient safety by reducing interruptions and flow variations^30^.

**Fig. 12:**
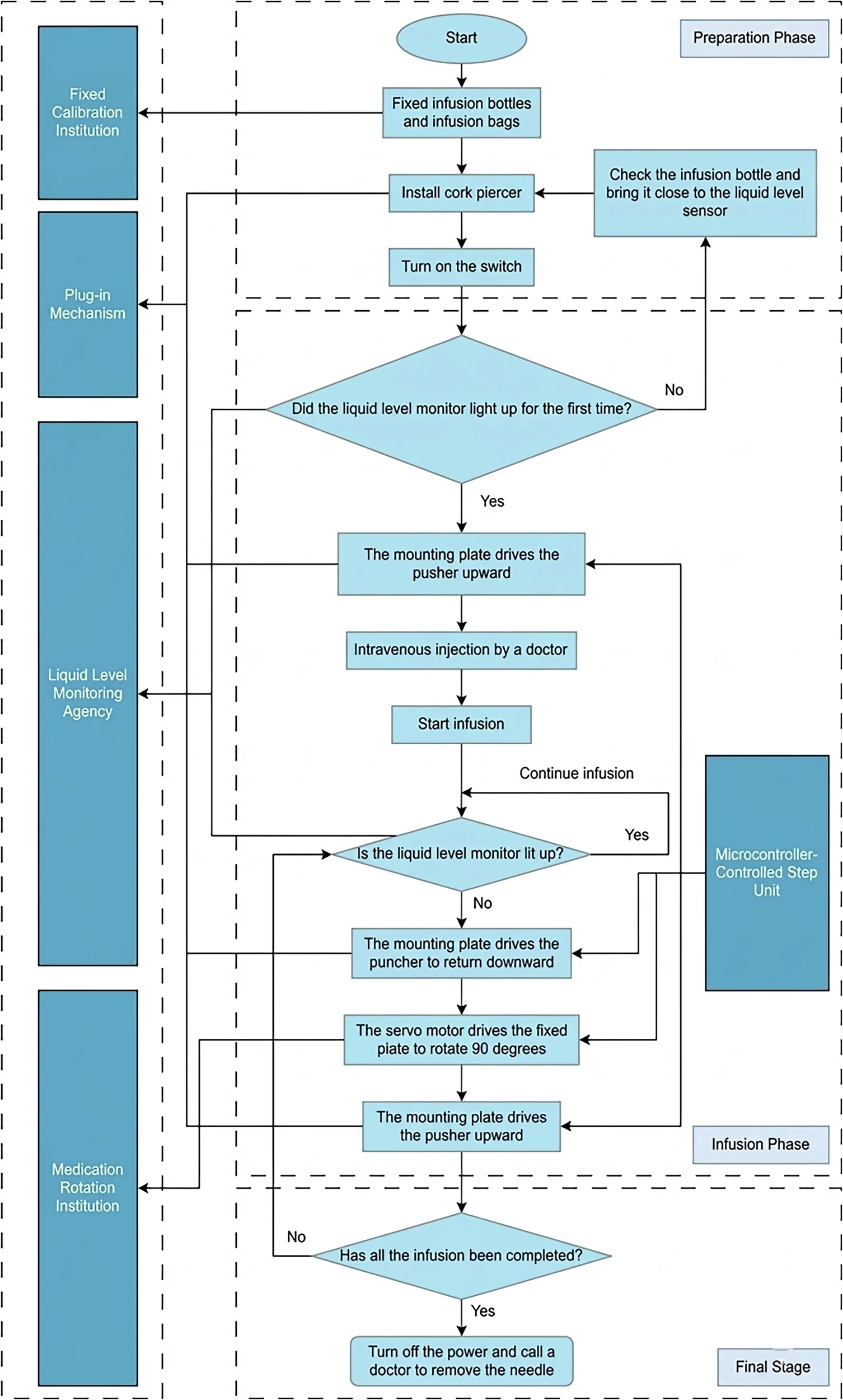
Operational workflow diagram of the smart IV infusion automatic medication replacement device illustrating the automated fluid monitoring and bottle-switching sequence^30^. To begin with the infusion bottle (typically an intermediate bulk container) workflow, the primary input is through a liquid level detector which provides fluid level data to a microcontroller every second, and initiates an automation cycle based upon the results of that data. When liquid is at a low level (typically approximately 35–40 units of liquid), the microcontroller sends out a pulse which will retract the in-use piercer from the depleted bottle, the rotary actuator will move to the new full bottle, and then insert the piercer into the new bottle, allowing the infusion process to continue automatically. All of this occurs without any involvement on the part of the nurse at the bedside, consequently providing uninterruptible drug delivery to the patient. As a result of the ability to automatically initiate a cycle of continual IV therapy, the risks associated with gap in care are reduced, the time the nurse spends responding to change-in-use requests are decreased, and the risk of air embolism due to delay in changing bottles is minimized. Additionally, because the automation workflow provides one centralized location to monitor several patients simultaneously, the nursing staff will be able to monitor multiple patients from one location rather than visiting each patient’s bedside.

Figure 12 presents an automated IV infusion system that monitors fluid levels and autonomously replaces empty bottles to ensure continuous therapy ^30^.

As shown in Fig. 12 the IV infusion system is an automated setup that improves safety, efficiency, and continuous patient treatment. It uses a microcontroller and a liquid level sensor to detect when the infusion bottle is empty, then it automatically removes the piercer, switches to a new bottle, and restarts the infusion process. This helps keep the therapy running without interruption and reduces the need for manual work by healthcare staff. It also lowers the risk of problems like air embolism and allows clinicians to focus more on caring about patients^30^.

**Fig. 13:**
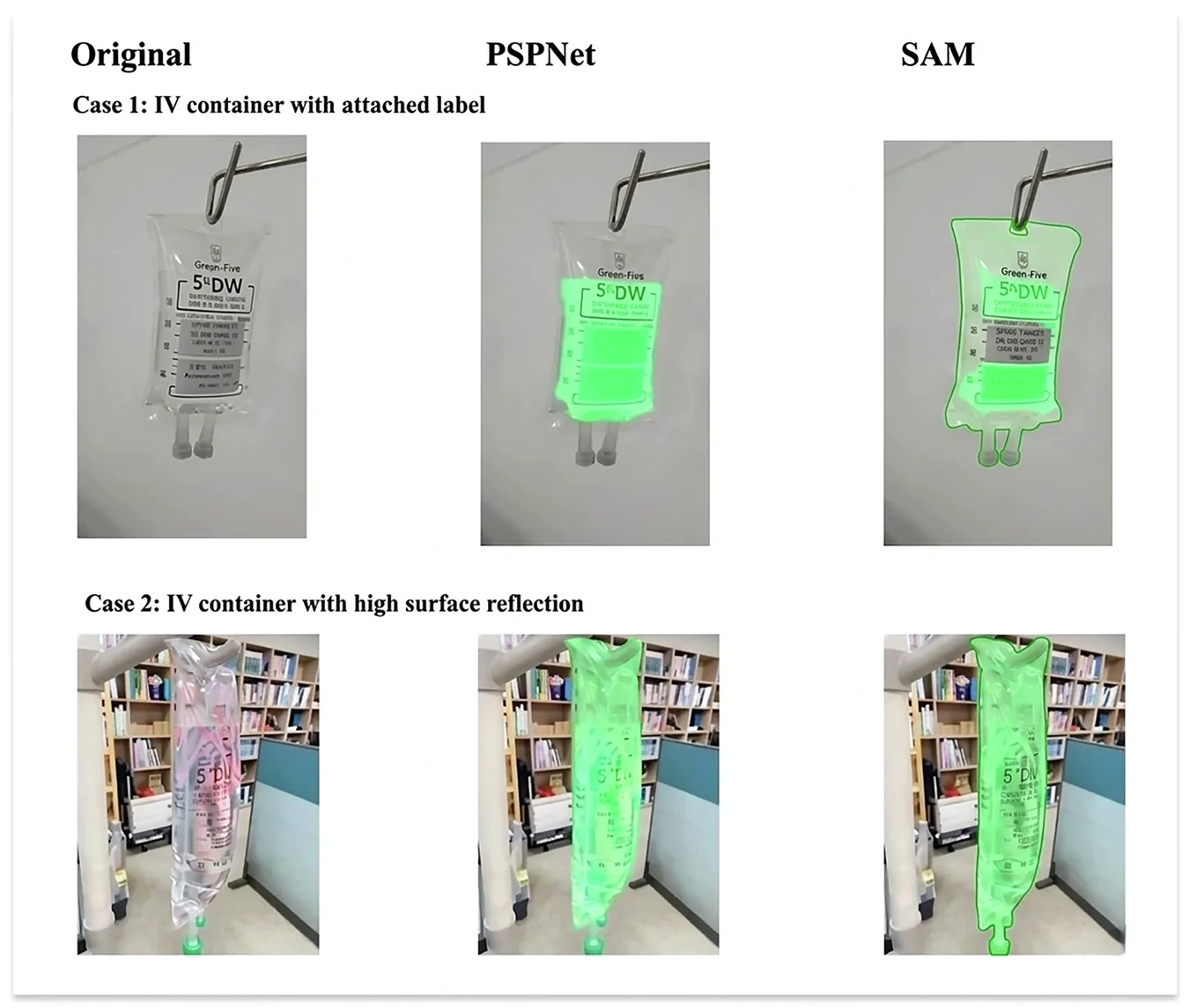
Visual comparison of Pyramid Scene Parsing Network and Segment Anything Model performance for IV fluid segmentation under challenging container conditions including label occlusion and surface reflections^34^. Each row in the figure presents one test case, showing from left to right: the original IV container image, the segmentation output produced by the Pyramid Scene Parsing Network (PSPNet), and the segmentation output produced by the SAM. Two representative challenging conditions are illustrated: cases where container labels partially occlude the fluid region, and cases where strong surface reflections interfere with optical fluid boundary detection. Both conditions indicate that PSPNet is more accurate than SAM in defining the area of fluid by recognizing the fluid boundary even when there are visual disturbances. The results obtained from SAM are highly sensitive to reflectivity and occluded labels, leading to incomplete segmentation masks. These results support the suitability of PSPNet as a preferred computer vision approach for automated IV fluid level monitoring in clinical environments where container appearance variability is unavoidable.

Figure 13 compares liquid segmentation performance using Pyramid Scene Parsing Network (PSPNet) and Segment Anything Model (SAM) on IV containers under challenging conditions such as labels and reflections^34^.

Figure 13 illustrates the liquid identification results of PSPNet and SAM when faced with difficult IV container conditions. For each case, an original picture, PSPNet output and SAM output has been provided. Also included are examples of each instance that contain labels and have a strong surface reflection. Results of this test shows that in both instances, PSPNet has demonstrated a greater level of accurate and robust liquid identification than SAM^34^.

**Fig. 14:**
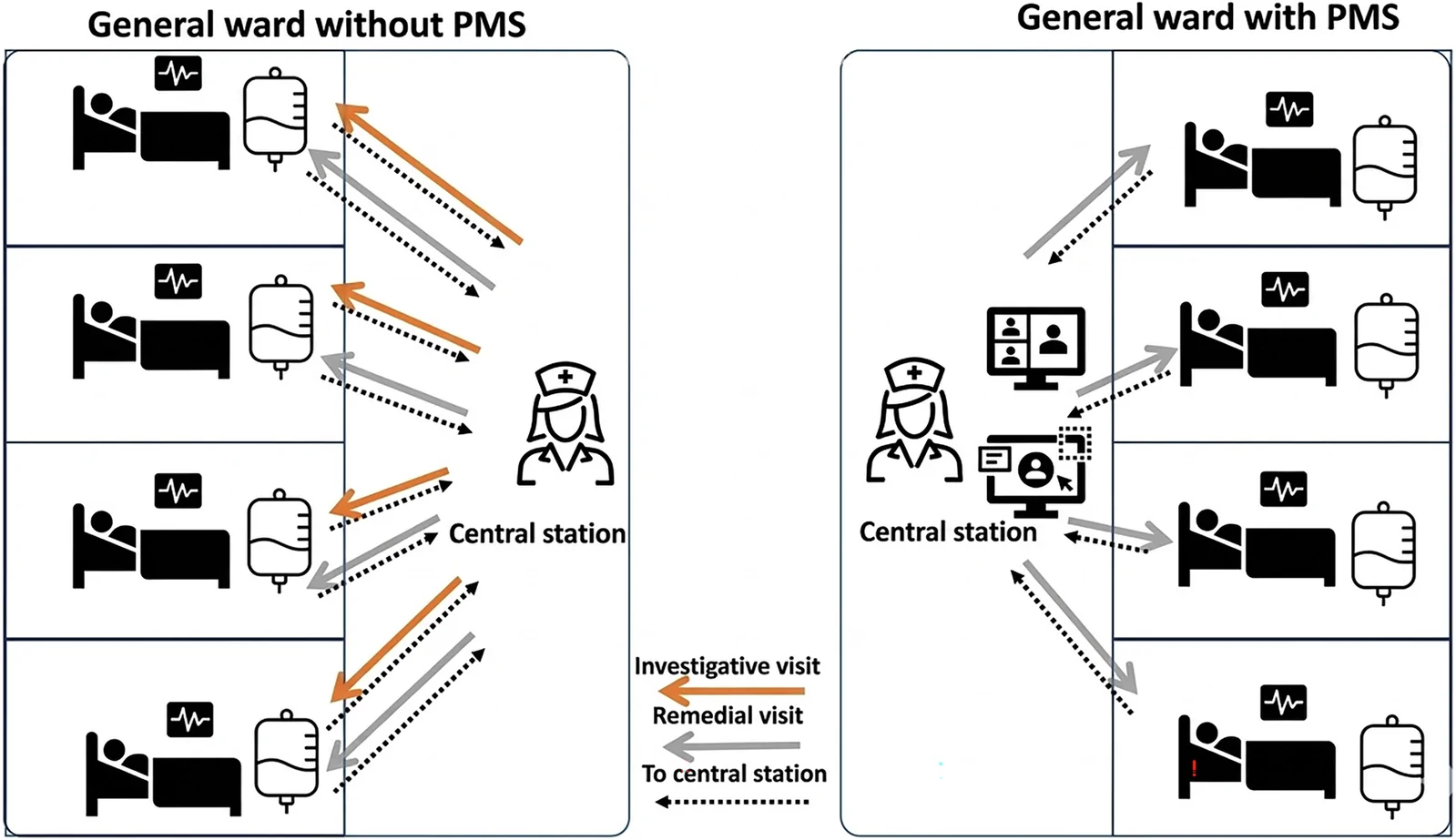
Comparative study of nursing procedures in general wards with/without a centralized Patient Monitoring System in relation to nurse mobility, monitoring, and response effectiveness^58^. Left diagram represents a standard ward setting without a PMS. Nurses need to personally visit each patient’s bed to verify the status of the infusion pump and the health status of the patient, which consumes a lot of their time and does not allow them to simultaneously monitor other patients. In contrast, the right diagram represents the same ward with a PMS, where the patient’s vital signs data and the status of the infusion pump are automatically transmitted to the central nursing station. Thus, only one nurse can monitor multiple patients at once. The setup saves nursing staff from making unnecessary physical rounds and decreases the time to respond to clinical incidents, allowing the nurses to engage in direct nursing tasks.

Figure 14 compares ward operations with and without a PMS, highlighting differences in workflow and efficiency^58^.

Figure 14 shows a hospital ward with and without a PMS. Without PMS, nurses have to go to each patient bedside to check infusion pumps and patient conditions, which takes a lot of time and effort. With PMS, patient data and infusion status are sent to a central station, so nurses can monitor many patients at once. This makes the ward more efficient, reduces unnecessary checks, and improves patient safety by allowing faster response when needed^58^.

## Discussion

### General Trends

The literature reviewed indicates that there is now a convergence of AI, IoT and wearable technology used in health care for continuous monitoring, infusion management, and early warning systems^1,8,12^. In every study reviewed, there appears to be a movement away from isolated sensing devices toward integrated digital eco-systems consisting of biosensors, ML, and wireless communications; thus, creating real-time decision support capabilities^1,40,54^. However, a comparative analysis reveals that most wearable systems are more mature in terms of continuous monitoring^14,69–82^, while IV infusion technologies are still largely focused on basic automation and alert generation rather than intelligent decision-making^24,26,53^. Smart infusion systems primarily focus on safety features such as flow control and alarm systems, while wearable devices dominate in terms of performance focus as long-term patient monitoring and predictive analytics^22,69,70,83^, whereas smart infusion systems primarily emphasize safety features such as flow control and alarm systems^30,84–86^. This indicates an imbalance in technological maturity, where wearable technologies are generally more advanced in AI integration^15,17,22^ compared to infusion systems, which remain more hardware-centric and rule-based^25,87,88^.

### Relation to INFUSYNC

From the perspective of INFUSYNC, the literature highlights a clear gap between monitoring systems and therapeutic delivery systems. Wearable technologies such as smart wristbands explain strong capability in continuous physiological tracking ^22,69,70^, while smart infusion systems mainly operate in isolation with limited intelligence and weak contextual awareness^89–91^. Although IoT-based infusion pumps provide basic connectivity and alerting functions^57,84,89^, they lack integration with real-time physiological feedback loops^27,55^, unlike wearable systems that increasingly incorporate AI-driven analytics^15,17,22^. This separation indicates that current approaches are fragmented rather than unified^3,9,12^, and no existing system fully bridges patient monitoring and infusion control in a closed, synchronized architecture^19,27,55^. Therefore, INFUSYNC addresses a critical gap by attempting to merge these two domains, enabling bidirectional interaction between patient state monitoring and infusion decision-making^31,56,69^. This positions the framework as a more holistic alternative compared to existing single-domain solutions^26,29,32^.

### Challenges, limitations, and deployment readiness

Regardless of the significant advances in the literature, different persistent limitations still obstruct real-world clinical implementation. First, most AI-based IV infusion systems rest at the prototype or experimental phase with limited clinical validation^33–35,55^^,^ while wearable technologies are more mature due to wider real-world adoption and testing^14,69,70^. This clearly shows an imbalance in development maturity between monitoring devices and therapeutic infusion systems. Second, interoperability remains a major challenge. Very few systems support integration with hospital infrastructures such as EHR systems or standardized communication protocols like HL7 FHIR^56,59,92^, which limits their ability to function within real clinical workflows^9,49,93,94^. As a result, most solutions still operate as isolated systems rather than fully connected hospital platforms. Security is another critical but underdeveloped area. None of the reviewed IV infusion systems include strong, built-in data protection mechanisms^28,30,84^, despite handling highly sensitive patient information. Although advanced security solutions such as blockchain, FL and encryption frameworks are widely discussed in broader IoT healthcare research ^48,60–62^^,^ they are rarely implemented in actual infusion or wearable systems, highlighting a clear gap between theory and practice^49,65^. In addition, closed-loop control is still largely missing in most IV systems^84,85,89^. Current solutions mainly rely on alert-based systems^57,89,91^, where clinicians must manually respond to events, rather than systems automatically adjusting infusion rates. Only a few studies, such as^55^, attempt closed-loop control using DL, but these remain in laboratory settings without real clinical deployment^27,55^. This creates a gap between AI potential and real hospital implementation, where human engagement is still required in most situations^56,58,59^.

Overall, the analysis shows that wearable systems are closer to clinical readiness due to higher maturity and adoption ^14,69,70^, while IV infusion systems still lag behind in intelligence, interoperability, and autonomy^31,55,57^. This clear inequality highlights the need for integrated frameworks such as INFUSYNC that unify sensing, decision-making, and infusion control into a single clinically achievable system^19,20,45^.

### Future directions and system-level perspective

The future direction of smart healthcare is clearly moving toward integrated ecosystems that integrate wearables, infusion systems, and cloud-based analytics^12,40,54^. However, the literature suggests that most current research still enhance individual components rather than full system integration^3,9,10,49^. As a result, scalability and clinical approval remain limited^4,8,95^. For INFUSYNC, this highlights the significance of not only integrating technologies but also ensuring clinical validation^14,70^, security by design^48,60,62^, and interoperability with hospital infrastructure^56,59,92^. Future systems must move beyond monitoring toward predictive and autonomous healthcare models^19,45,46^ that reduce clinician workload while maintaining safety^21,23,47^. In conclusion, the reviewed studies indicate that while enabling technologies are rapidly progressing across all clusters^1,12,54^, their integration remains the key obstacle^3,8,9^. INFUSYNC is positioned to address this gap by unifying wearable monitoring and infusion automation into a single intelligent, secure, interoperable, and clinically validated framework^31,48,55,56,69^.

## Conclusion

This paper reviewed the convergence of wearable wristband monitoring, smart infusion automation, and continuous patient monitoring in preceded safer and more intelligent healthcare systems. The findings prove that AI, IoT, and wearable technologies are not only emerging concepts but have already achieved clinically validated performance levels in several domains. For example, AI-based monitoring systems have reported high accuracy with HR error of ±1.1 bpm, SpO₂ error of ±0.72%, and RR error of ±0.84 breaths per minute (*r* = 0.97)^69^, while wearable IoT devices have shown uniformly low measurement variations within ±1.83 bpm for HR, ±1.2% for SpO₂, and ±0.15 °C for temperature^70^. These results confirm that the most important sensing and analytics technologies are already achieving clinical-grade consistency. Importantly, obverse to the perception that key challenges remain unresolved, the literature provides diverse validated solutions addressing main system limitations. Security and privacy concerns have been actively tackled using blockchain-enabled FL^48^, permissioned blockchain frameworks^60^, secure EHR integration models^61^, and advanced encryption-based architectures^62^, all of which demonstrate experimentally validated effectiveness rather than theoretical proposals. Similarly, problems related to sensor instability and noise have been mitigated through in-sensor processing techniques^15^ and robust ML ensembles^22^. Furthermore, interoperability with hospital systems has already been demonstrated in clinical environments through HL7-compliant smart pump integrations in ICU and pediatric settings^56,59,92^, confirming that integration with EHR workflows is technically achievable. Therefore, the major restriction is not the absence of solutions, but rather the fragmentation of existing validated technologies across separate domains. Current studies largely validate wearable systems, infusion systems, and security frameworks independently, but fail to evaluate their combined performance within a unified clinical environment. No study to date has simultaneously assessed wearable bio-signal monitoring, closed-loop IV infusion control, and secure interoperable communication under real hospital conditions, leaving an important gap in system-level validation rather than component-level capability. The INFUSYNC framework directly addresses this integration gap by combining these already-validated technologies into a unified architecture and evaluating them within the same clinical workflow. This shifts the focus from proposing separate solutions to integrating and clinically validating existing high-maturity technologies in an arranged system. The proposed INFUSYNC implementation further strengthens this direction by enabling real-world testing of synchronized wearable monitoring and infusion automation.

Future work should therefore move beyond repeating isolated technological improvements and instead prioritize: (1) multi-site clinical validation of integrated systems under real workflow conditions, (2) mandatory HL7 FHIR-based interoperability as a design standard, and (3) systematic deployment of already-validated blockchain and encryption frameworks within unified healthcare architectures. Addressing integration and system-level validation, rather than re-proposing already-solved component-level challenges, will be the key step toward translating these technologies into safe, scalable, and clinically deployable healthcare solutions^66^.

## Data Availability

No datasets were generated or analysed during the current study.
